# Mass Spectrometry Quantification Revealed Accumulation of C-Terminal Fragment of Apolipoprotein E in the Alzheimer's Frontal Cortex

**DOI:** 10.1371/journal.pone.0061498

**Published:** 2013-04-11

**Authors:** Meiyao Wang, Illarion V. Turko

**Affiliations:** 1 Institute for Bioscience and Biotechnology Research, Rockville, Maryland, United States of America; 2 Biomolecular Measurement Division, National Institute of Standards and Technology, Gaithersburg, Maryland, United States of America; Institute of Enzymology of the Hungarian Academy of Science, Hungary

## Abstract

Polymorphic variation in the apolipoprotein E (apoE) gene is the major genetic susceptibility factor for late-onset Alzheimer's disease (AD) and likely contributes to neuropathology through various pathways. It is also recognized that apoE undergoes proteolytic cleavage in the brain and the resultant apoE fragments likely have a variety of bioactive properties that regulate neuronal signaling and may promote neurodegeneration. ApoE fragmentation in the human brain has been intensively studied using different immunochemical methods, but has never been analyzed in a quantitative manner to establish preferably accumulated fragments. Here we report quantification using multiple reaction monitoring mass spectrometry (MRM MS) with ^15^N-labeled full-length apoE4 as an internal standard. Measurements were performed on frontal cortex from control and severe AD donors. Our data point to a preferable accumulation of C-terminal apoE fragment in the insoluble fraction of tissue homogenate in the severe AD group versus the control group. Further insight into the biological consequences of this accumulation may lead to a better understanding of the basic mechanism of AD pathology.

## Introduction

Apolipoprotein E (apoE) is a glycoprotein that was initially recognized for its importance in lipid transport in peripheral circulation and the central nervous system. More recently, it has been studied for its role in Alzheimer's disease (AD). Human apoE has three major isoforms, apoE2, apoE3, and apoE4, which differ by single amino acid substitutions involving cysteine-arginine replacements at positions 112 and 158 [1].ApoE4 (*ε*4 allele) is a major genetic risk factor for late-onset AD [2]. Although numerous studies support a role of apoE4 in enhancing the risk for AD, the mechanism by which apoE4 contributes to AD pathogenesis remains obscure. Several lines of evidence point to the fact that apoE4 is highly susceptible to proteolysis compared to apoE3, and that the link between apoE4 and AD may be in generation of stable fragments that enhance pathology.ApoE (≈34 kDa) contains two domains, referred to as the N-terminal (≈ 22 kDa) and C-terminal (≈10 kDa) domains, which are connected by a short hinge region [3]. The hinge has multiple protease-sensitive sites and can be cleaved by a wide range of proteases [3]–[6]. The N-terminal domain is thought to harbor the low-density lipoprotein receptor binding site while the C-terminal domain is believed to mediate apoE oligomerization and contains the major lipid binding site. First, analysis of proteins co-purified with amyloid fibrils revealed≈8–9-kDa C-terminal fragments of apoE [7]. Later, a 22 kDa N-terminal fragment of apoE was found in brain and cerebrospinal fluid [8]. In addition, fragments generated by off-hinge cleavage were also reported. These include a 30 kDa N-terminal fragment (carboxyl-terminal-truncated apoE), which is generated by cleavage at sites within the C-terminal domain [4], [9], and an 18 kDa N-terminal fragment, which is generated by cleavage within the N-terminal domain [4], [9], [10]. Overall, presence of N- and C-terminal apoE fragments in brain extracts was confirmed by immunoblotting with N-terminal-specific and C-terminal-specific antibodies [11]. Despite intensive research on apoE fragments [5], [12]–[15], there is no consensus on which fragments are preferably accumulated and what their actual pathological functions are, if any. The relative distribution between soluble and insoluble cell fractions also remains unclear. Furthermore, it has not yet been determined whether formation of fragments is dependent on the particular apoE isoforms or presence of AD.

We have recently reported a mass spectrometry-based approach for selective quantification of apoE isoforms [16]. In the present study, we utilized this approach to assess protein level and soluble/insoluble distribution of apoE based on N- and C-terminal peptides of total apoE and apoE4 isoform in the control and AD frontal cortex from human postmortem brain. For the first time, we provide quantitative evidence for a preferable accumulation of apoE C-terminal fragment in the insoluble fraction of AD frontal cortex homogenate.

## Methods

### Materials

Ammonium chloride (^15^N, 99%) was purchased from Cambridge Isotope Laboratories (Andover, MA). The *DC* Protein Assay kit was from Bio-Rad Laboratories (Hercules, CA). Sequencing grade modified trypsin was obtained from Promega Corp. (Madison, WI). All other chemicals were purchased from Sigma-Aldrich (St. Louis, MO).

### Expression and Purification of ^15^N-Labeled apoE4

The plasmid containing human cDNA for full-length apoE4 in pET32a vector was kindly provided by Dr. Karl Weisgraber (Gladstone Institute of Neurological Disease, UCSC, San Francisco, CA). ^15^N-Labeled apoE4 (^15^N-apoE4) was expressed as a His-tagged apoE4-thioredoxin fusion protein in One Shot BL21(DE3) competent *E.coli* (Invitrogen, Grand Island, NY) as described [16]. Initial purification of ^15^N-apoE4 on Ni-NTA Agarose column (Qiagen, Valencia, CA) and further purification on a hydroxyapatite (Bio-Rad Laboratories, Hercules, CA) column were performed as described [16]. The purification resulted in a protein with molecular mass≈50 kDa as determined by SDS PAGE. In-gel trypsin digestion in combination with mass spectrometry analysis on a 4700 Proteomics Analyzer (AB Sciex, Framingham, MA) confirmed the protein as a ^15^N-apoE4-thioredoxin fusion protein, further referred to as ^15^N-apoE4.

### Human Tissues

Samples of frontal cortex were received from the Washington University School of Medicine Alzheimer's Disease Research Center. Human tissues were collected in accordance with a guidance of the Washington University Human Research Protection Office (HRPO number: 89-0556). The authors consulted the HRPO, which determined that Institutional Review Board (IRB) oversight was not required for this study. In the state of Missouri, individuals can give prospective consent for autopsy. Our participants provide this consent by signing the hospital's autopsy form. If the participant does not provide future consent before death, the DPOA or next of kin provide it after death. All data were analyzed anonymously. Demographic information on the donors is summarized in Table S1. The Clinical Dementia Rating (CDR) listed in Table S1 is a numeric scale used to quantify the severity of symptoms of dementia. The composite score of CDR ranging from 0 through 3 with CDR equals 0 for no symptoms and CDR equals 3 for severe symptoms (http://www.biostat.wustl.edu/~adrc/cdrpgm/index.html).

### Sample Processing

Sample processing followed the previously published protocol [16] with minor modifications. In brief, the human brain tissue was placed in 25 mmol/L NH_4_HCO_3_ and homogenized by sonication on ice at 30 W using three 10 s continuous cycles (Sonicator 3000, Misonix Inc., Farmingdale, NY). The total protein concentration was measured in the presence of 1% SDS (mass concentration) using the *DC* Protein Assay kit and bovine serum albumin as a standard. The homogenates were then aliquoted into 0.2 mg-portions of total tissue protein *per* tube; this is referred to as the whole tissue homogenate. One set of tubes was frozen at −80°C while another set of tubes was first centrifuged at 153,000 × g for 30 min to generate the high-speed supernatant and pellet, which were separately frozen at −80°C. Consequently, the measurements of total apoE and specific apoE4 were later performed on three different sets of samples: the whole tissue homogenate, the high-speed supernatant, and the high-speed pellet. In all cases, the samples were placed in 25 mmol/L NH_4_HCO_3_/1% SDS/20 mmol/L DTT and supplemented with 1 pmol of ^15^N-apoE4. The mixture was incubated at room temperature for 60 min to allow reduction of cysteines and was then treated with 50 mmol/L iodoacetamide for another 60 min. Alkylated samples were precipitated with chloroform/methanol [17]. The protein pellets obtained were sonicated in 100 µL of 25 mmol/L NH_4_HCO_3_ and treated with trypsin for 15 h at 37°C. The substrate/trypsin ratio was 50:1 (weight/weight). After trypsinolysis, 0.5% trifluoroacetic acid (TFA) (volume concentration) was added to each sample. The samples were then centrifuged at 153,000 × g for 30 min and the peptide-containing supernatants were transferred to new tubes and dried using a Vacufuge (Eppendorf AG, Hamburg, Germany).

### MRM MS Analysis

The dried peptides were reconstituted in 3% acetonitrile/0.1% formic acid (volume concentrations). Peptides separation and MRM analysis were performed on a hybrid triple quadrupole/linear ion trap mass spectrometer (4000 QTRAP, AB Sciex, Framingham, MA) coupled to an Eksigent nanoLC-2D system (Dublin, CA). Peptides were separated and eluted at a flow rate of 300 nL/min over 30 min-gradient of acetonitrile from 15% to 35% containing 0.1% formic acid in H_2_O by an Eksigent cHiPLC- nanoflex system equipped with a nano cHiPLC column, 15 cm × 75 µm, packed with ReproSil-Pur C18-AQ, 3 µm (Dr. Maisch, Germany). The eluted sample was directed into the nanospray source of the mass spectrometer controlled by Analyst 1.5.1 (AB Sciex, Framingham, MA). The subsequent MRM detection of signature peptides was performed in the positive ion mode with the following major parameters: an ion spray voltage of 2200 V, curtain gas of 15 psi, source gas of 20 psi, interface heating temperature of 170 °C, declustering potential of 76 V for +2 precursor ions and 65 V for +3 precursor ions, collision cell exit potential of 16 V for +2 precursor ions and 13V for +3 precursor ions, and dwell time of 40 ms.

### Data Analysis

Initial list of MRM transitions was selected as described [18] and was experimentally screened for the three most intensive transitions per peptide. These transitions were further used for quantification and are listed in the Table S2. The relative ratios of the three transitions monitored in the 25 mmol/L NH_4_HCO_3_ for ^15^N-apoE4 were similar to those observed by spiking ^15^N-apoE4 into the human brain samples. This confirms no significant interference from tissue for the quantification based on the selected transitions. The linearity of the optimum transitions was verified by spiking whole homogenate samples with varying amounts of the ^15^N-apoE4. The identities of the measured peptides were confirmed based on the retention time of the three MRM peaks from a given peptide and the relative ratio among the three MRM peaks. Protein concentrations were calculated from the ratio of the light and heavy MRM peak intensities multiplied by the known amount of ^15^N-apoE4 spiked into the sample. All three transitions from each peptide were treated as independent measurements, each resulting in a concentration value expressed as *pmol* of the quantified protein per *mg of tissue protein*. The level of each given peptide was based on the mean±SD of the transitions from this peptide.The consensus apoE concentrations were calculated as a mean±SD based on selected signature peptides.

## Results and Discussion

### Full-length Protein Standard

**Figure 1:**
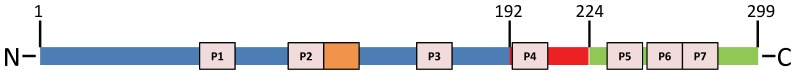
Schematic representation of apoE. The N-terminal domain (22 kDa), hinge, and C-terminal domain (10 kDa) are shown in *blue*, *red*, and *green*, respectively. Signature peptides used to quantify apoE based on the N-terminal domain (P1, P2, and P3), hinge (P4), or C-terminal domain (P5, P6, and P7) are shown as *pink* boxes. A unique peptide used to selectively quantify the apoE4 isoform is located in the N-terminal domain and shown as an *orange* box.

Figure 1 provides a schematic representation of apoE protein. The N-terminal domain, hinge, and C-terminal domain are shown in *blue*, *red*, and *green*, respectively. Isotope-labeled, full-length protein standard (^15^N-apoE4) allows simultaneous quantification of a total apoE based on different peptides derived from the N-terminal domain, hinge, or C-terminal domain and therefore these measurements should all result in the same quantification so long as apoE has not been fragmented. However if cleavage of hinge occurred and accumulation of either domain exceeds another one, the quantitative differences based on individual peptides will appear. In addition, ^15^N-apoE4 has a unique peptide (shown as an *orange* box), which allows selective quantification of a combined full-length and N-terminal domain of apoE4 isoform.

### Quantification of Total ApoE

**Figure 2:**
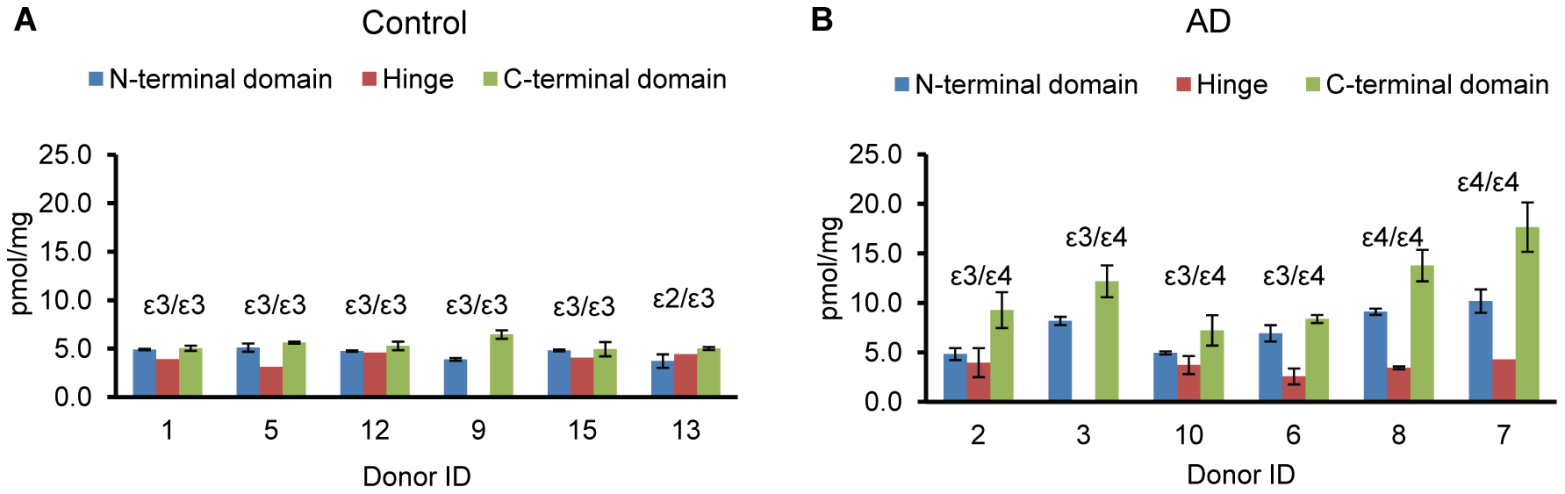
Quantification of total apoE in whole frontal cortex homogenate. Measurements were performed on the control (A) and severe AD (B) frontal cortex. The apoE genotype is shown for each donor. The concentration of total apoE in pmol/mg total protein was calculated based on peptides in the N-terminal, hinge, and C-terminal domains and is color coordinated. The concentration was calculated for three experimental replicates by monitoring three transitions per individual peptide. For N-terminal and C-terminal domain the data for three signature peptides were combined. All final data are presented as the mean±SD.

We have quantified total apoE based on three peptides in the N-terminal domain (P1, P2, and P3), one peptide in the hinge (P4), and three peptides in the C-terminal domain (P5, P6, and P7). The amino acid sequences and MRM transitions for these peptides are summarized in Table S2. Measurements were performed for whole homogenates of frontal cortex from control and severe AD donors and three transitions were monitored *per* peptide. For each donor, measurements based on P1, P2, and P3 showed less than 20% variation (Table S3) and were combined into a single mean value for the N-terminal domain. Measurements for each donor based on P5, P6, and P7 also showed less than 20% variation (Table S3) and were combined into a single mean value for the C-terminal domain. In other words, each quantification of total apoE, one based on the N-terminal domain and one based on the C-terminal domain, represents an average of three transitions *per* peptide for three peptides *per* domain. The summarized data for whole homogenate are plotted as a bar graph in Figure 2.

It appears that measurements based on the hinge peptide P4 were consistently lower than measurements based on the N- and C-terminal domains. These lower values were especially obvious for the AD group (Figure 2B). Given that the hinge region is prone to cleavage by multiple proteases, it prompted a conclusion that hinge region of apoE undergoes more efficient cleavage in AD group versus control group. However, this is not the only one explanation for lower measurements based on hinge peptide P4. It was reported that Thr^212^ in the hinge peptide P4 (AAT^212^VGSLAGQPLQER) is O-glycosylated in the apoE from human cerebrospinal fluid [19]. Data for potential hinge glycosylation in brain are not available. Nevertheless, there appear to be different overlapping conditions that might result in lower measurements based on hinge peptide P4 observed in the current study.

We have to emphasize that quantification based on the N-terminal domain represents a combined amount of the full-length apoE plus N-terminal fragment(s), if any. The same is correct for the quantification based on the C-terminal domain, which represents a combined amount of the full-length apoE plus C-terminal fragment(s), if any. It means that we cannot state the existence of fragments based on these quantifications taken alone. However, comparison of quantifications based on N-terminal and C-terminal domains can demonstrate which fragment is accumulated in excess. The quantification of apoE based on the N-terminal domain shows low donor-to-donor scatter and was from 3.8 to 5.2 pmol apoE/mg of tissue protein in the control group. The quantification of apoE based on the C-terminal domain also showed low donor-to-donor scatter in the control group; however, the absolute values were consistently a little higher in a range from 5.0 to 6.8 pmol apoE/mg of tissue protein. In the AD group, the difference between quantification based on the N-terminal and C-terminal domain was apparent. The concentrations were in a range from 4.8 to 9.1 pmol apoE/mg tissue protein based on the N-terminal domain and substantially higher from 8.0 to16.0 pmol apoE/mg tissue protein based on the C-terminal domain.

Taken together, our quantitative data recognize higher total level of apoE in the AD group. However, these data can also be interpreted as an accumulation of both apoE fragments, N-terminal and C-terminal, since our quantifications represent a combined amount of full-length apoE and either one fragment. Overall, the evident observation from these measurements is that for the first time quantitative data point to a preferable accumulation of apoE C-terminal fragment in the AD group versus control group.

**Figure 3:**
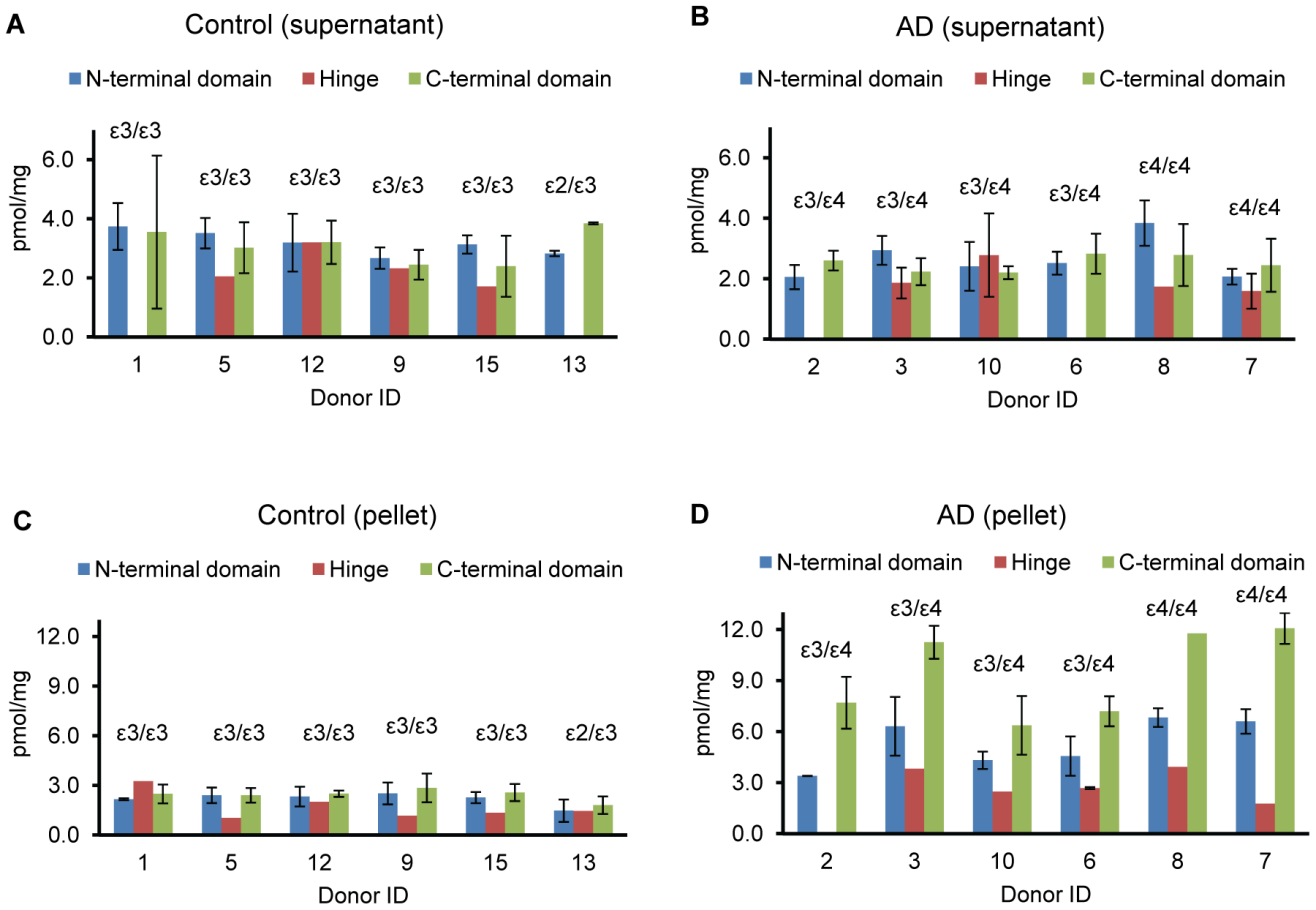
Quantification of total apoE in supernatant and pellet fractions of whole frontal cortex homogenate. Measurements were performed on the control (A and C) and severe AD (B and D) frontal cortex. The supernatant (A and B) and pellet (C and D) fractions were generated by centrifugation of whole frontal cortex homogenate at 153,000 × g for 30 min. The concentration of total apoE in pmol/mg total protein was calculated based on peptides in the N-terminal, hinge, and C-terminal domains and is color coordinated. The concentration was calculated for three experimental replicates by monitoring three transitions per individual peptide. For N-terminal and C-terminal domain the data for three signature peptides were combined. All final data are presented as the mean±SD.

Tissue apoE can be separated into two pools by high-speed centrifugation, with soluble apoE in the supernatant and insoluble apoE in the pellet. The apoE recovered from the pellet most likely represents apoE oligomers and/or apoE complexes with biological membranes and amyloid beta [5], [7], [11]. We have tracked C-terminal fragment accumulation by quantifications in the supernatant and pellet fractions obtained by high-speed centrifugation of the whole homogenate. Measurements were performed on the same samples of frontal cortex from the control and severe AD donors and summarized in Figure 3. Measurements in the supernatant based on N- and C-terminal domains demonstrated similar levels of total apoE in both control and AD groups (Figure 3A and 3B). This indicates that the pool of soluble apoE does not have preferable accumulation of either fragment. In contrast, measurements in the pellet based on the C-terminal domain were higher than those based on the N-terminal domain in AD group (Figure 3D). This demonstrated accumulation of the C-terminal fragment in the pellet of AD group versus control group where measurements based on both domains were similar (Figure 3C). In summary, we have assigned the AD-associated C-terminal fragment accumulation to the pellet fraction of whole tissue homogenate.

### Quantification of apoE4 and Data Analysis Based on apoE Genotype

ApoE4 was measured based on apoE4-specific peptide (LGADMEDVR) located in the N-terminal domain (shown in *orange* in Figure 1). Measurements for apoE4 were performed in-parallel with total apoE in the same MRM runs. The goal of selective apoE4 quantification was to explain the difference (if any) between apoE3 and apoE4 in the C-terminal domain accumulation. Table 1 shows combined data for three apoE genotypes; *ε*3/*ε*3 (5 control donors, n = 5), *ε*3/*ε*4 (4 AD donors, n = 4), and *ε*4/*ε*4 (2 AD donors, n = 2). It is important to underline that the data for apoE4 in *ε*4/*ε*4 genotype samples based on common N-terminal peptides (9.7±1.0 pmol/mg tissue protein) and based on specific apoE4 peptide (10.0±1.0 pmol/mg tissue protein) are almost identical (Table 1). This comparison validates consistency of apoE and apoE4 MRM measurements based on the ^15^N-apoE4 internal standard.

**Table 1:** Comparison of total apoE and apoE4 quantifications.

	ApoE, pmol/mg tissue protein					
	whole homogenate		supernatant		pellet	
	N-terminal	C-terminal	N-terminal	C-terminal	N-terminal	C-terminal
Control (*ε3*/*ε3*)	4.7±0.5	5.5±0.7	3.3±0.7	2.9±1.1	2.3±0.4	2.6±0.5
AD (*ε3*/*ε4*)	6.2±1.5	9.2±2.1	2.5±0.6	2.5±0.5	4.8±1.4	8.0 ±2.1
AD (*ε4*/*ε4*)	9.7±1.0	15.7±2.8	3.0±1.1	2.6±0.8	6.7±0.5	12.0±0.8

	ApoE4, pmol/mg tissue protein		
	whole homogenate	supernatant	pellet
AD (*ε3*/*ε4*)	3.1±0.8	1.5±0.7	2.2±0.5
AD (*ε4*/*ε4*)	10.0±1.0	3.0±0.4	7.4±0.3

We further calculated the ratio of concentrations obtained based on C-terminal versus N-terminal domains. In the supernatant, these ratios deviate little from 1.0. At the same time, these ratios gradually increase in the whole homogenate for *ε*4/*ε*4 (1.62)>*ε*3/*ε*4 (1.48)>*ε*3/*ε*3 (1.17) and in the pellet for *ε*4/*ε*4 (1.79)>*ε*3/*ε*4 (1.67)>*ε*3/*ε*3 (1.13). To find a significant difference between sample groups, we calculated a proportion between ratios and *P*-value. The pair-wise comparison is summarized in Table 2 and shows a significant *P*-value (≤ 0.05) for comparison of AD *vs.* control and *ε*4/*ε*4 *vs. ε*3/*ε*3. It seems that this could point to apoE4 as a source of C-terminal fragment accumulation. However, it is important to remember that the sample size of the *ε*4/*ε*4 group is small and this interpretation has a limitation since all of the *ε*3/*ε*3 samples are in the control group while all of the *ε*3/*ε*4 and *ε*4/*ε*4 samples are in the AD group.

**Table 2:** Pair-wise comparison of the ratios of C-terminal domain vs. N-terminal domain quantification between sample groups.

sample groups	whole homogenate		pellet	
	proportion	*P*-value	proportion	*P*-value
AD *vs.* control	1.3	0.05	1.6	0.001
(*ε3*/*ε4*) *vs.* (*ε3*/*ε3*)	1.3	0.13	1.6	0.029
(*ε4*/*ε4*) *vs.* (*ε3*/*ε3*)	1.4	0.06	1.6	0.015
(*ε4*/*ε4*) *vs.* (*ε3*/*ε4*)	1.1	0.61	1.0	0.996

## Conclusions

We have provided quantitative data that are consistent with earlier observations in the field of apoE fragmentation. This includes the AD-associated higher level of total apoE, which also could be interpreted as accumulation of the N-terminal and C-terminal apoE fragments. In addition, for the first time, we have provided quantitative data about the AD-associated preferable accumulation of apoE C-terminal fragment. Furthermore, we have assigned the accumulation of C-terminal fragment to the insoluble pool of apoE, which represents a pellet fraction of the whole homogenate after high-speed centrifugation. Our initial data suggest that apoE4 could be a source of the C-terminal fragment accumulation; however other experimental approaches and larger sample size will be worthy for its validation as well as for further investigation of functional consequences of C-terminal fragment accumulation.

## Supporting Information

Table S1 Information on the donors of frontal cortex.
(DOCX)

Table S2 Peptides and MRM transitions used for the quantification.
(DOCX)

Table S3 Total apoE concentrations quantified in the whole homogenate based on individual P1, P2, P3, P4, P5, P6, and P7 peptides.
(DOCX)
