# Supplementary material for: Mass Spectrometry Quantification Revealed Accumulation of C-Terminal Fragment of Apolipoprotein E in the Alzheimer's Frontal Cortex
Source: PLoS One. 2013 Apr 11;8(4):e61498. doi: 10.1371/journal.pone.0061498 (PMC3623866; doi:10.1371/journal.pone.0061498)
Supplement: Table S1 — Information on the donors of frontal cortex. (DOCX) [file pone.0061498.s001.docx]

**Table S1. Information on the donors of frontal cortex.**

| Donor ID | Age (y) | Gender | Clinical Dementia Rating |
| --- | --- | --- | --- |
| 1 | 90 | F | 0 |
| 5 | 87 | M | 0 |
| 9 | 95 | F | 0 |
| 12 | 81 | F | 0 |
| 13 | 87 | M | 0 |
| 15 | 91 | M | 0 |
| 2 | 79 | M | 3 |
| 3 | 74 | F | 3 |
| 6 | 80 | F | 3 |
| 7 | 82 | F | 3 |
| 8 | 81 | F | 3 |
| 10 | 91 | F | 3 |
