# Supplementary material for: Mass Spectrometry Quantification Revealed Accumulation of C-Terminal Fragment of Apolipoprotein E in the Alzheimer's Frontal Cortex
Source: PLoS One. 2013 Apr 11;8(4):e61498. doi: 10.1371/journal.pone.0061498 (PMC3623866; doi:10.1371/journal.pone.0061498)
Supplement: Table S2 — Peptides and MRM transitions used for the quantification. (DOCX) [file pone.0061498.s002.docx]

|  | MRM transitions (*m/z*) | | | | |
| --- | --- | --- | --- | --- | --- |
|  | Protein/Peptides | Q1 (charge) | Q3 (type, charge) | | |
| apoE4 | LGADMEDVR | 503.24 (+2) | 892.38 (y^8^,+1) | 835.36 (y^7^,+1) | 649.30 (y^5^,+1) |
|  | ^15^N- LGADMEDVR | 509.22 (+2) | 903.35 (y^8^,+1) | 845.33 (y^7^,+1) | 657.27 (y^5^,+1) |
| P1 | SELEEQLTPVAEETR | 577.62 (+3) | 801.41 (y^7^, +1) | 704.36 (y^6^,+1) | 605.29 (y^5^,+1) |
|  | ^15^N- SELEEQLTPVAEETR | 583.93 (+3) | 811.38 (y^7^, +1) | 713.33 (y^6^,+1) | 613.27 (y^5^,+1) |
| P2 | ELQAAQAR | 443.74 (+2) | 644.35 (y^6^,+1) | 516.29 (y^5^,+1) | 445.25 (y^4^,+1) |
|  | ^15^N-ELQAAQAR | 450.22 (+2) | 655.32 (y^6^,+1) | 525.26 (y^5^,+1) | 453.23 (y^4^,+1) |
| P3 | LAVYQAGAR | 474.77 (+2) | 764.41 (y^7^, +1) | 665.34 (y^6^,+1) | 502.27 (y^5^,+1) |
|  | ^15^N-LAVYQAGAR | 481.25 (+2) | 775.37 (y^7^, +1) | 675.31 (y^6^,+1) | 511.25 (y^5^,+1) |
| P4 | AATVGSLAGQPLQER | 499.94 (+3) | 642.36 (y^5^,+1) | 545.30 (y^4^,+1) | - |
|  | ^15^N-AATVGSLAGQPLQER | 506.59 (+3) | 651.33 (y^5^,+1) | 553.28 (y^4^,+1) | - |
| P5 | EQVAEVR | 415.72 (+2) | 557.26 (b^5^,+1) | 573.34 (y^5^, +1) | 474.27 (y^4^, +1) |
|  | ^15^N -EQVAEVR | 421.21 (+2) | 563.24 (b^5^,+1) | 581.31 (y^5^, +1) | 481.25 (y^4^, +1) |
| P6 | LEEQAQQIR | 557.80 (+2) | 872.46 (y^7^, +1) | 743.42 (y^6^,+1) | 615.36 (y^5^,+1) |
|  | ^15^N -LEEQAQQIR | 565.27 (+2) | 885.42 (y^7^, +1) | 755.38 (y^6^,+1) | 625.33 (y^5^,+1) |
| P7 | LQAEAFQAR | 517.28 (+2) | 721.36 (y^6^,+1) | 592.32 (y^5^,+1) | 521.28 (y^4^,+1) |
|  | ^15^N -LQAEAFQAR | 524.25 (+2) | 731.33 (y^6^,+1) | 601.29 (y^5^,+1) | 529.26 (y^4^,+1) |

**Table S2. Peptides and MRM transitions used for the quantification.**
