# Supplementary material for: Mass Spectrometry Quantification Revealed Accumulation of C-Terminal Fragment of Apolipoprotein E in the Alzheimer's Frontal Cortex
Source: PLoS One. 2013 Apr 11;8(4):e61498. doi: 10.1371/journal.pone.0061498 (PMC3623866; doi:10.1371/journal.pone.0061498)
Supplement: Table S3 — Total apoE concentrations quantified in the whole homogenate based on individual P1, P2, P3, P4, P5, P6, and P7 peptides. (DOCX) [file pone.0061498.s003.docx]

|  | ApoE, pmol/mg tissue protein | | | | | | |
| --- | --- | --- | --- | --- | --- | --- | --- |
|  | N-terminal domain | | | hinge | C-terminal domain | | |
|  | P1 | P2 | P3 | P4 | P5 | P6 | P7 |
| Donor ID |  |  |  |  |  |  |  |
| 1 | 4.68 ± 0.87 | 4.96 ± 0.56 | 4.85 ± 0.74 | 3.90 ± 0.29 | 4.85 ± 0.48 | 5.49 ± 0.77 | 5.23 ± 1.07 |
| 5 | 4.67 ± 1.03 | 5.52 ± 0.55 | 5.16 ± 0.39 | 3.13 ± 0.23 | 5.70 ± 0.36 | 5.45 ± 1.06 | 5.55 ± 0.76 |
| 12 | 4.83 ± 2.38 | 4.70 ± 0.55 | 4.73 ± 0.47 | 4.58 ± 0.31 | 5.59 ± 0.82 | 5.85 ± 0.91 | 4.97 ± 1.15 |
| 13 | 4.21 ± 1.15 | 4.03 ± 1.12 | 2.92 ± 0.39 | 4.44 ± 0.27 | 5.13 ± 1.37 | 5.26 ± 1.10 | 4.92 ± 0.66 |
| 9 | 3.80 ± 1.14 | 3.82 ± 1.45 | 4.05 ± 0.77 | - | 6.87 ±0.66 | 6.53 ± 0.24 | 6.00 ± 0.36 |
| 15 | 4.92 ± 0.78 | 4.76 ± 1.50 | 4.79 ± 0.60 | 4.07 ± 0.27 | 4.11 ±0.62 | 5.25 ± 1.64 | 5.49 ± 1.35 |
|  |  |  |  |  |  |  |  |
| 2 | 6.19 ± 0.48 | 5.26 ± 0.52 | 4.40 ± 0.79 | 3.96 ± 1.47 | 10.55 ± 0.92 | 9.20 ± 0.82 | 7.99 ± 1.31 |
| 3 | 6.15 ± 0.69 | 7.89 ± 1.58 | 8.47 ± 0.09 | - | 13.32 ± 1.88 | 9.31 ± 0.63 | 11.05 ± 1.83 |
| 8 | 9.06 ± 0.90 | 8.82 ± 1.37 | 9.46 ± 1.67 | 3.44 ±0.15 | 12.65 ± 1.17 | 15.70 ± 1.19 | 14.90 ± 0.74 |
| 10 | 5.00 ± 0.63 | 5.08 ± 0.78 | 4.78 ± 1.66 | 3.72 ± 0.91 | 6.13 ± 0.53 | 9.00 ± 1.06 | 8.30 ± 0.44 |
| 6 | 6.17 ± 1.54 | 6.82 ± 1.22 | 7.79 ±1.31 | 2.57 ± 0.81 | 8.84 ± 0.85 | 8.08 ± 1.14 | 8.23 ± 1.40 |
| 7 | 8.82 ± 0.99 | 10.79 ± 1.53 | 10.95 ± 1.68 | 4.29 ± 0.19 | 15.90 ± 4.69 | 27.15 ± 2.18 | 19.42 ± 1.44 |

**Table S3. Total apoE concentrations quantified in the whole homogenate based on individual P1, P2, P3, P4, P5,**

**P6, and P7 peptides.**
